# Rare cell isolation and recovery on open-channel microfluidic chip

**DOI:** 10.1371/journal.pone.0174937

**Published:** 2017-04-20

**Authors:** Taisuke Masuda, Woneui Song, Hayao Nakanishi, Wu Lei, Anas Mohd Noor, Fumihito Arai

**Affiliations:** 1 Department of Micro-Nano Systems Engineering, Graduate School of Engineering, Nagoya University, Nagoya, Japan; 2 Laboratory of Pathology and Clinical Research, Aichi Cancer Center Aichi Hospital, Nagoya, Japan; The Ohio State University, UNITED STATES

## Abstract

The ability to accurately detect and analyze rare cells in a cell population is critical not only for the study of disease progression but also for next flow cytometry systems in clinical application. Here, we report the development of a prototype device, the ‘Rare cell sorter’, for isolating and recovering single rare cells from whole blood samples. On this device, we utilized an open-channel microfluidic chip for rare cell isolation. And the advantage of open-channel allows us to recover the isolated rare cell directly from the chip. We set the circulating tumor cell (CTC) as a target cell.

For the clinical experiment, CTCs were isolated from blood samples collected from patients with metastatic breast cancer and healthy volunteers. There was a significant difference in the number of CTCs between the patients with metastatic breast cancer and healthy volunteers. To evaluate the damage to cells during isolation and recovery, we performed an RNA integrity assay using RNA extracted from CTCs recovered from the chip and found that our process for single CTC isolation and recovery is mild enough for gene analysis of CTCs.

## Introduction

Isolation of rare cells (low-abundance cells), such as circulating tumor cells (CTCs) [1], fetal nucleated red blood cells (fNRBC) [2], and vascular epithelial cells (ECs) [3], from large population of background cells such as blood has a wide range of applications. Several studies have analyzed the genetic mutations carried by CTCs, comparing the mutations to those of primary tumors or correlating the findings to the severity or spread of the patient’s disease [4, 5]. Therefore, the leading applications of CTC analyses are real-time genetic analyses of tumor cells. This is a subject that has become critical in the new era of genetically targeted cancer therapies. Thus, peripheral blood might serve as a perfect alternative sample for cancer diagnoses, such that the analysis of CTCs has been termed ‘liquid biopsy’ [6, 7].

A further challenge for CTC researchers is the difficulty of colleting CTCs at the single-cell level. In recent years, the importance of single-cell analysis has grown rapidly for various fields, including drug discovery and regenerative medicine [8, 9]. Because of the inhomogeneous states in a cellular cluster, the analysis results suggest only serves average states. In contrast, some of mechanisms cannot be explained by average states because it depends on a threshold factor or stochastic component [10–12]. The determination of the true mechanism requires analysis of the cluster at the single-cell level to avoid the loss of information associated with ensemble averaging.

Even though researchers have developed many types of microfluidic chips for isolating CTCs [13], progress is hampered by cell loss associated with tube connections and difficulties in recovering isolated CTCs at the single-cell level, because most microfluidic chips are closed systems. To extract isolated target rare cell, the cell must move through the channel of the chip or tube. And it causes cell loss because it is difficult to keep the cell in the users view.

The blood sample to be used for CTC isolation is often treated with Ficoll-Paque or lysis buffer to remove mononucleocytes and red blood cells because higher cell concentrations (about 1×10^7^ cells/ mL) might clog the system and affect resolution [14]. However, these pre-treatments can result in significant cell loss and damage [15].

Our research on rare cell isolation and recovery for single-cell analysis has led to the development of a prototype device with microfluidics called the ‘Rare cell sorter’. The ‘Rare cell sorter’ achieves isolation and direct recovery (pick-up) of rare cells from low-pretreated whole blood for single-cell analysis. For these purposes, we utilized an open-channel microfluidic chip and micropipette manipulation. Basically, CTCs, our main target, are isolated from blood sample on the open-channel microfluidic chip based on their size compared to blood cells. After isolation, individual CTCs can be recovered by micropipette manipulation. Our open-channel microfluidic chip has a high efficiency for CTC isolation and all the processes, including isolation and recovery, can be operated with less damage.

## Materials and methods

### Rare cell sorter

**Fig 1:**
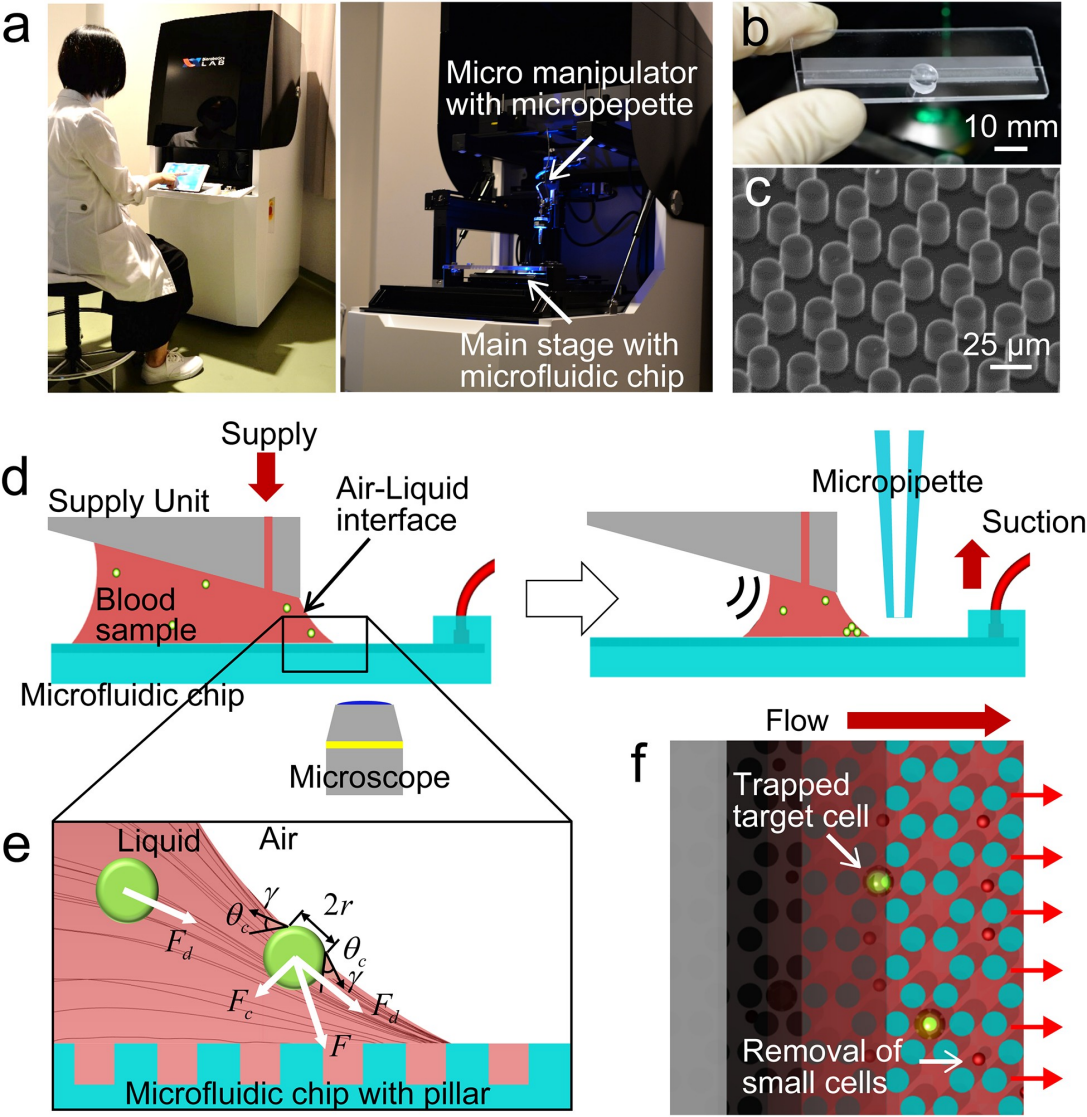
Overview of the ‘Rare cell sorter’ for isolating and recovering single rare cells from whole blood samples. (a) External view of the ‘Rare cell sorter’. (b) Fabricated open-channel microfluidic chip. The opaque area is the main pattern with micropillars. (c) Scanning electron microscope image of the main pattern. (d) Concept of the open-channel microfluidic chip for CTC isolation and recovery. The blood sample is introduced between the supply unit and the open-channel microfluidic chip. Because of gravitational potential energy, the remaining sample gathers along a side, which has a small gap between the supply unit and microfluidic chip. Isolated CTCs can be recovered by micropipette manipulation. (e) Schematic illustration of the forces acting on the CTCs during the isolation process. The results of finite element analysis of fluid dynamics in the blood sample show that CTCs move toward the meniscus of the air–liquid interface along the black lines, which represent streamlines resulting from the drag force (*Fd*). The CTCs are then subjected to capillary force (*Fc*) associated with the meniscus of the air–liquid interface. As a result, CTCs are trapped on the microfluidic chip due to the total force (*F*), comprising *Fd* and *Fc*. (f) CTCs drift toward the meniscus of the air–liquid interface and are trapped on the microfluidic chip. Small cells, such as red blood cells (RBCs), pass through the gaps between pillars, while CTCs and some white blood cells (WBCs) remain trapped due to their larger size.

Fig 1a shows the ‘Rare cell sorter’. As shown in Fig 1b, a core part of the device is the open-channel microfluidic chip. The main pattern on the chip is the open channel. Fig 1c shows a scanning electron microscope (SEM) image of the main pattern. The ‘Rare cell sorter’ also includes an optical system for CTC detection and motorized stages for manipulating the open-channel microfluidic chip and micropipette. All of these components are mounted inside a custom-designed case and can be operated using an iPad graphic user interface. The method of CTC isolation is shown in Fig 1d, which presents a schematic illustration of the research concept. First, a diluted whole blood sample is introduced between a supply unit and the open-channel microfluidic chip and held there. The blood sample needs to be diluted with the same volume of PBS (EDTA-2Na solution) to avoid clogging. Next, the blood sample is aspirated through the microfluidic channels using a syringe pump. A pressure gradient is generated with a syringe pump. When the pump aspirates the blood sample through the chip, the blood sample is held between the chip and the supply unit as a result of capillary forces. For using this capillary force, the chip must be hydrophilic and it has a distance limit according to the flow rate. For our experiment (flow rate = 10~20 mL/hr), the distance limit was about 2mm. By this process, cells in the sample are carried to the meniscus of the air–liquid interface (Fig 1e). Cells within the blood sample, including CTCs, are then trapped in pockets on the microfluidic chip due to capillary force associated with the meniscus of the air–liquid interface [16]. Due to their relatively small size, blood cells pass through the gaps between micropillars and are flushed away, isolating the CTCs on the microfluidic chip (Fig 1f and S1 Movie). During the isolation process, the supply unit has a slope relative to the microfluidic chip so that the remaining blood sample is retained by gravitational potential energy toward the side that has a small gap between the supply unit and the microfluidic chip as the volume of blood sample decreases. Therefore, CTCs are trapped on a limited area of the microfluidic chip because the air–liquid interface remains stationary.

The forces acting on CTCs during the isolation process are illustrated in Fig 1e and can be classified as body, surface, and contact forces. CTCs floating in the blood sample are initially acted upon by gravity (*F*_*g*_) and buoyancy (*F*_*b*_) as volume forces. However, the effects of gravity and buoyancy are minimal because the density of the CTCs is approximately the same as that of the medium of the blood sample. At the beginning of the isolation process, CTCs are subjected to drag as a surface force associated with the flow of the blood sample. The drag force (*F*_*d*_) acting on the CTCs in the flow can be expressed by Stokes’ Law: *F*_*d*_ = 6*πηRv*, where *η* represents the viscosity of the blood sample suspension, *R* represents CTC radius, and *v* represents the velocity of the CTCs relative to the fluid.

We also confirmed the flow by finite element analysis (COMSOL Multiphysics 4.3, COMSOL Co., Ltd., Burlington, MA, USA). The results of the analysis suggested that CTCs move toward the meniscus of the air–liquid interface as a result of the drag force (*F*_*d*_) and then move along the meniscus. From the meniscus of the air–liquid interface, the CTCs are acted upon by capillary force (*F*_*c*_) associated with surface tension at the line of contact with the meniscus. The capillary force (*F*_*c*_) can be expressed as follows: *F*_*c*_ = 2*πrγ sin θ*_*c*_, where *r* represents the radius of the contact surface of the CTC, *γ* represents surface tension, and *θ*_*c*_ represents the contact angle between the meniscus of the air–liquid interface and the CTCs. The capillary force (*F*_*c*_) on the CTCs is oriented vertical to the meniscus of the air–liquid interface. In summary, the total force (*F*) acting on each CTC is composed of drag force (*F*_*d*_) and capillary force (*F*_*c*_) components, as illustrated in Fig 1e. The CTCs in the blood sample are pulled down toward the microfluidic chip because *F* acts in that direction.

CTCs trapped on the open-channel microfluidic chip can be visually detected with a fluorescence microscope and then recovered at the single-cell level using the micropipette manipulation. For this purpose, the micropipette is attached perpendicular to the microfluidic chip because we use an inverted microscope to prevent interference between the microscope’s lens and the micropipette.

### Design of open-channel microfluidic chip

Generally, CTCs are 15±10 μm in diameter [17, 18], whereas red blood cells (RBCs) and white blood cells (WBCs) are 6 to 8 and 10 to 12 μm in diameter, respectively [19]. We designed the shape of the microfluidic chip based on this information to isolate trapped cells according to size. Micropillars are arranged in a hexagonal pattern across the entire microfluidic chip surface, with one micropillar forming the vertex of each hexagon (S1 Fig). The space surrounded by the micropillars forms a pocket for trapping the CTCs. The most important dimension is the distance between two micropillars (W_P_) within a hexagon, as this distance determines the rates of both trapping CTCs and removing blood cells. Therefore, we constructed three different microfluidic chips, with W_P_ values of 6, 7, or 8 μm. The corresponding micropillars were 20, 18, and 16 μm in diameter (ϕ_P_), so that the trapping pocket diameter for each chip (ϕ_Pocket_) was approximately the same (32 μm). The height of the fluid channel (H_P_) was 30 μm across the entire microfluidic chip. The microfluidic chip was made with poly-dimethylsiloxane (PDMS) and fabricated basically by soft photolithography and dry etching of the silicon substrate. Entire microfluidic chip is designed to be wide (75 mm) because the CTC isolation throughput is proportional to the width of the microfluidic chip. The microfluidic chip with 75 mm-width allows to isolate 5 mL of whole blood sample in 30–60 min. From the preliminary experiment, 7 μm was found to be the most efficient W_P_, and 18 μm was chosen as the diameter of the micropillars in S3 Fig.

### Mouse CTC model

To evaluate the performance of our method, we used a mouse CTC model [20, 21] as a preclinical model. The mice were 7- to 8-week-old male athymic nude mice of the KSN strain (Japan SLC Inc., Hamamatsu, JPN) and housed in specific pathogen-free (SPF) conditions at the Aichi Cancer Center Research Institute. GCIY-EGFP cells, from the GFP-tagged gastric cancer cell line, were injected into mice subcutaneously, and tumor cells spontaneously generated lung metastasis at the macroscopic level 2–3 months after injection. The original GCIY (human gastric cancer cells) cell line is a poorly differentiated human gastric cancer cell line, established from ascites fluid (RIKEN Cell Bank, Tokyo, Japan). GCIY-EGFP cell line is a transfected GCIY cell line with the pEGFP-C1 plasmid (CLONTECH Lab. Palo Alto, CA) as described previously [22]

After sampling blood from mouse CTC models, images of the lung metastasis with GFP fluorescence were acquired using fluorescence field microscopy to observe the degree of metastasis. Using an image analyzer (ImageJ, NIH, Bethesda, MD, USA), the proportion of metastatic areas could be determined based on differences in intensity. We defined 50 a.u. as the intensity difference between metastatic and non-metastatic spots. Mouse CTC models were divided into high-metastatic and low-metastatic groups based on the proportion of metastatic areas on a 5% basis. Thus, five blood samples from each group were utilized for our main experiment. All animal experiments were performed under the experiment protocol approved by the Ethics Review Committee for Animal Experimentation of the Aichi Cancer Center and met the standard as defined by the United Kingdom Coordinating Committee on Cancer Research guidelines.

### Metastatic breast cancer patients and healthy volunteers

Characteristics of breast cancer patients; For the clinical experiment, 13 stage IV breast cancer patients with distant metastasis (M1), such as bone, lung, liver metastasis from the Department of Breast Oncology, Aichi Cancer Center Central Hospital and 10 healthy volunteers as negative controls were enrolled. The average age of each group was 45 and 31, respectively. Venous blood samples (5 mL) were collected in tubes containing EDTA-2Na and used for examination within 6 h. This study was approved by the institutional review board of the Aichi Cancer Center, and all participants provided written informed consent to participate in the study.

### Total RNA integrity assay of recovered CTCs

For gene analysis of CTCs, RNA recovered from isolated CTCs must be intact. For comparison of cells before and after recovery (including isolation), we used GCIY-EGFPs as CTC surrogates. For quantification of the RNA, an Agilent 2100 Bioanalyzer (Agilent Technologies Inc., Palo Alto, CA, USA) [23, 24], which achieves separation of charged biological molecules (RNA, DNA) based on the microfluidic chip, was used. The integrity of RNA can be assessed using the Bioanalyzer by visualization of the 28S and 18S ribosomal RNA bands. The degradation of RNA is indicative with an elevated threshold baseline and a decreased 28S:18S ratio [25]. The level of degradation is computed using a specific algorithm and returned as an RNA Integrity Number (RIN) from 1 to 10, with a larger RIN indicating more intact RNA.

## Results

### Isolation of CTCs from the blood sample of a mouse CTC model

**Fig 2:**
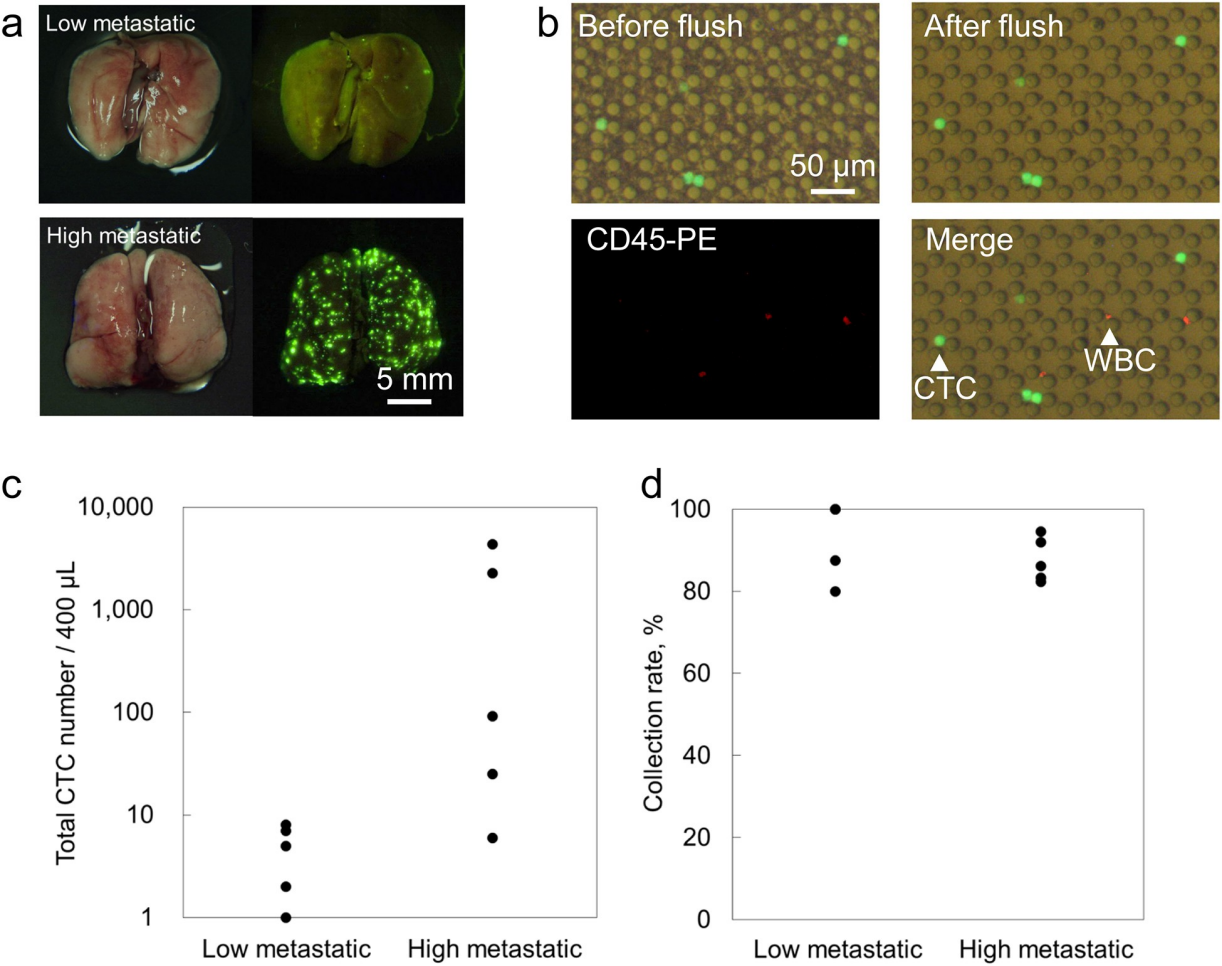
Detection of CTCs from the blood sample of a mouse CTC model. (a) Bright-field image and fluorescent image of the lungs of mouse CTC models. The models were classified into low- and high-metastatic groups by their degree of metastasis. (b) Images of the chip during the CTC isolation process. CTCs are trapped on the chip, and blood cells can be flushed away. White blood cells were stained with CD45-PE (red). (c) Total number of CTCs for each sample. Larger numbers of CTCs were confirmed in the high-metastatic group. (d) Collection rates for each sample. The average collection rates for the low- and high-metastatic groups were 93.5% and 87.7%, respectively.

We evaluated the use of the microfluidic chip using a mouse CTC model [20, 21]. The mouse CTC model is made by subcutaneous injection of GCIY-EGFPs into mice. The GCIY-EGFPs spontaneously metastasize to the lungs of the mice after 2–3 months. A blood sample (400 μL) was collected from each of 10 mouse CTC models for isolation of CTCs. Each mouse was classified into either a low- or high-metastatic group (Fig 2a). CTCs from the mouse CTC model basically express enhanced green fluorescent protein (EGFP), and before introduction of the sample, we stained the blood sample with CD45-PE for identification of WBCs (White blood cells). The total numbers of CTCs in each blood sample was counted under a fluorescent microscope by spreading on slide glasses before the isolation experiments. EGFP of CTCs were bright enough to be recognized. Fig 2b shows trapped CTCs and residual WBCs on the microfluidic chip during isolation and after flushing.

As shown in Fig 2c, the high-metastatic group contained a greater number of CTCs than the low-metastatic group. On average, there were 4.6 cells from the low-metastatic group and 1,350 cells from the high-metastatic group in the 400-μL mouse blood sample. Next, we evaluated the collection rate of CTCs (Fig 2d). Here, collection rate is the fraction of captured target cells in the region above the white dotted line (S1 Movie) relative to the number of target cells in the original sample. The microfluidic chip provided a high collection rate for CTCs for both the low-metastatic group (avg. 93.5%) and the high-metastatic group (avg. 87.7%). In summary, we could determine the correlation between the number of CTCs and the degree of metastasis. Furthermore, our microfluidic chip enabled us to isolate CTCs with a high collection rate for both high- and low-metastatic groups.

### Isolation of CTCs from the blood sample of cancer patients

We also evaluated the use of the microfluidic chip in clinical experiments with cancer patients. We examined blood sampled from 13 patients with metastatic breast cancer and 10 healthy volunteers for the presence of CTCs. The volume of each blood sample was 5 mL, and isolation processes took 30–60 min. After isolated from each blood sample on the chip, CTCs were identified based on the CD45-PE(-) / Hoechst33342(+) / Cytokeratin-AE1/AE3-Alexa 488(+) staining pattern. The staining process was done on the chip right after CTC isolation process. Each CD45-PE, Hoechst33342 and Cytokeratin-AE1/AE3-Alexa 488 identifies WBCs, nucleated cells and tumor cells.

**Fig 3:**
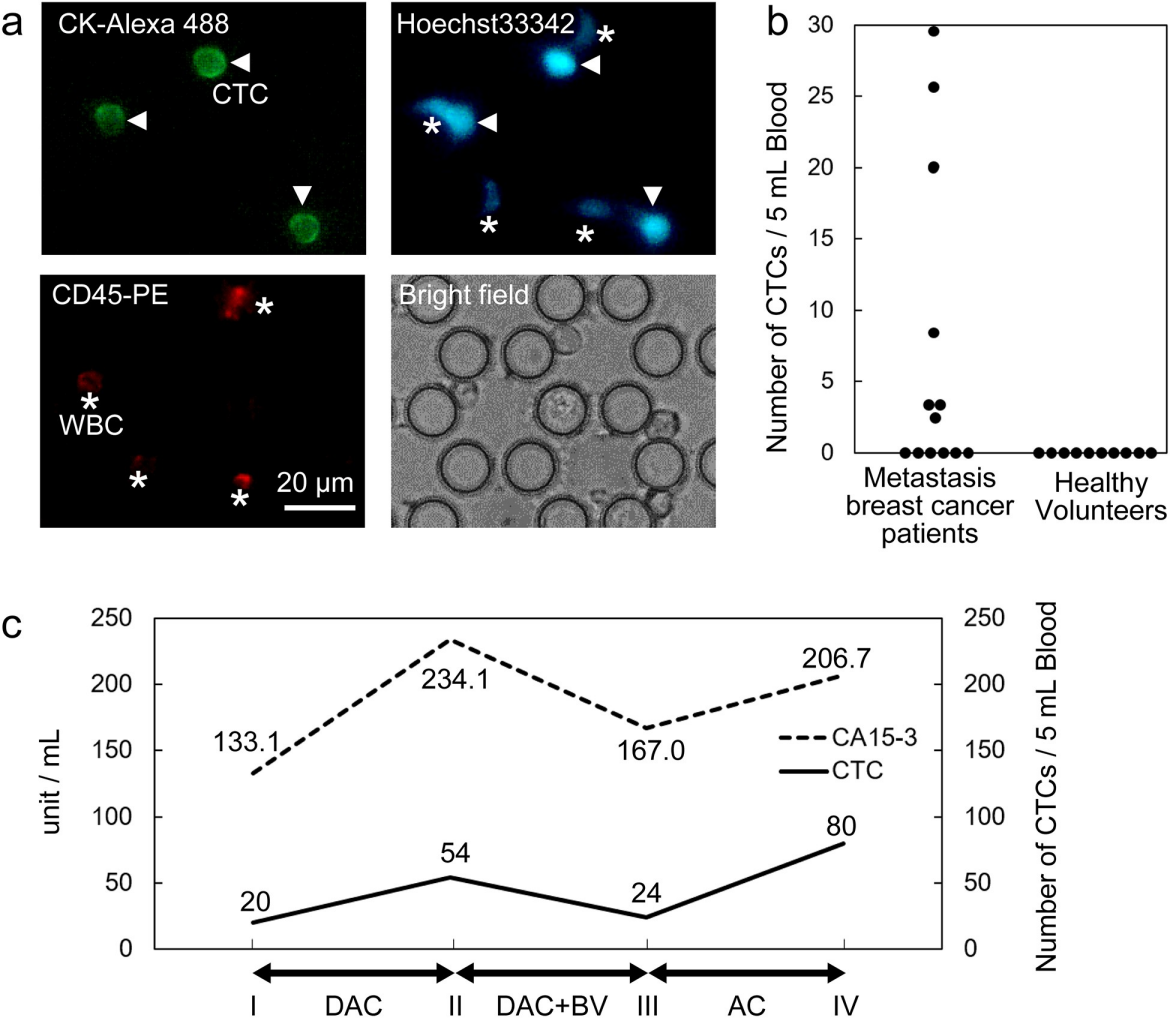
Detection of CTCs from a patient with metastatic breast cancer. (a) Images of CTCs from a patient with metastatic breast cancer. The sample was stained with Cytokeratin-AE1/AE3-Alexa 488, Hoechst33342, and CD45-PE. Both CTCs and WBCs are visible. (b) The number of CTCs for patients with metastatic breast cancer and healthy volunteers. An average of 7 CTCs were detected in the blood samples from 4 out of 10 patients with metastatic breast cancer while no CTCs were detected in the blood sample from the 10 healthy volunteers (c) Five-month comparison between the number of CTCs and serum CA15-3 levels from a patient with metastatic breast cancer. For each interval, the therapies were DAC, DAC+BV, and AC (DAC: dichloroacetate, BV: bevacizumab, AC: anthracycline + endoxan).

Fig 3a shows images of CTCs isolated from the blood samples of patients with metastatic breast cancer. As shown in the figure, relatively large WBCs can be trapped at the same time. However, CTCs can be recovered (picked-up) with minimum WBCs because the pockets are separated from each other.

Although the clinical experiment had a small sample number, the difference in the number of CTCs between metastasis breast cancer patients and healthy volunteers is significant, as shown in Fig 3b. CTCs were detected in the blood from 7 out of 13 patients (average number of CTCs = 7 per 5 mL) while no CTCs were detected in the blood from healthy volunteers. Moreover, we examined the results of CTC isolation four separate times over 5 months for one breast cancer patient and compared those results with serum levels of CA15-3 [26], which is a biomarker for breast cancer. The examinations were performed every time the therapy for the patient was changed. As shown in Fig 3c, there was a correlation between the number of CTCs and the serum CA15-3 level. Based on similar numerical changes in both the number of CTCs and the serum CA15-3 level, we confirmed the CTC isolation performance of our device and the usefulness of CTCs as a cancer biomarker.

### Recovery of single CTCs and evaluation of their RNA integrity

**Fig 4:**
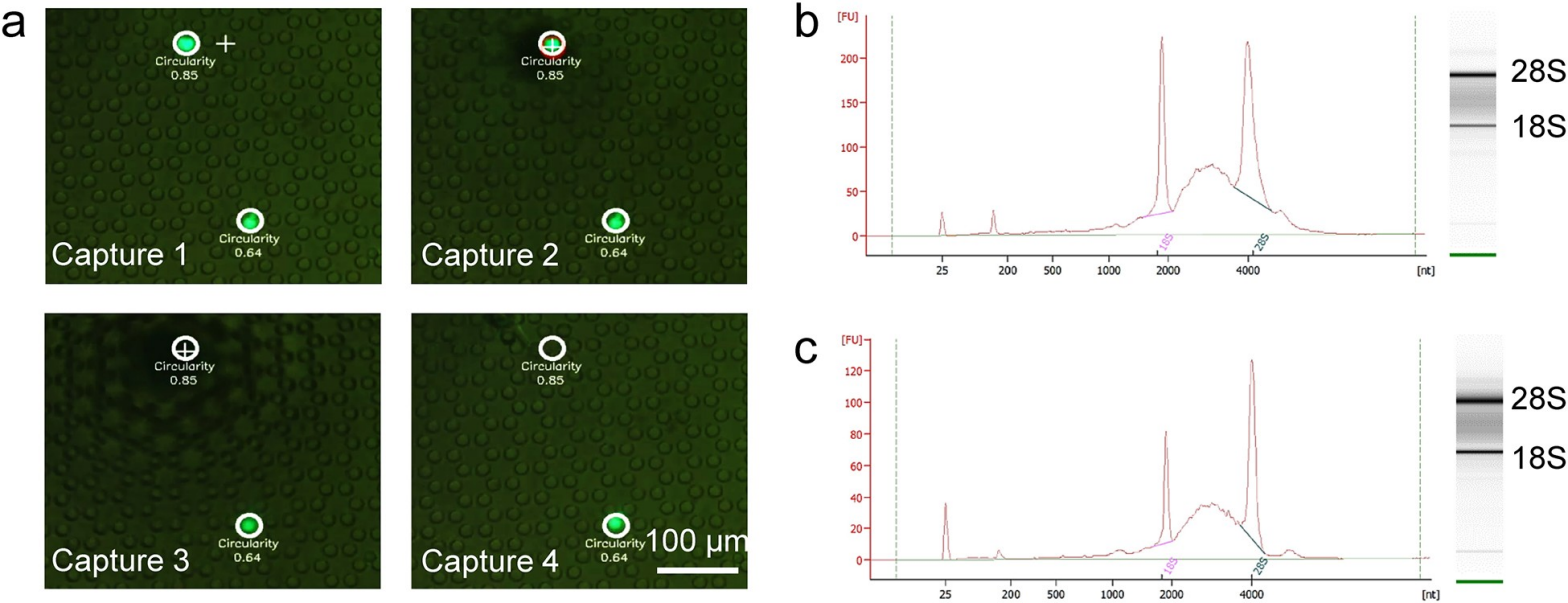
Single CTC recovery and RNA integrity. (a) Recovery process of isolated CTCs (capture1–capture4 in order). Isolated CTCs can be detected and some information can be obtained (e.g., circularity). A selected CTC is recovered by micropipette manipulation. And RNA integrity results before (b) and after (c) isolation and recovery with GCIY-EGFPs as a surrogates.

As explained above, our open-channel microfluidic chip allows direct recovery by micropipette manipulation (Fig 4a and S2 Movie). Moreover, to realize single-cell analysis, isolated and recovered CTCs must be intact. We evaluated changes in the integrity of total RNA of CTCs during isolation and recovery. For comparison between the before and after processes (isolation, recovery), we used GCIY-EGFPs as CTC surrogates.

The RIN of the GCIY-EGFPs was 8.0 (Fig 4b) before isolation and recovery, and declined slightly to 7.9 (Fig 4c) after isolation and recovery. Thus, gene analysis of CTCs is possible using our process for CTC isolation and recovery. For comparison, we left the GCIY-EGFPs for 5 h at room temperature, and the RIN declined to 2.9. Even though the RNA integrity depends on the original state of the CTCs, these results show that we can expect high RNA integrity of isolated (and recovered) CTCs.

## Discussion

Analysis of CTCs is an active areas of cancer research because these cells can be used as biomarkers for liquid biopsy to estimate the risk of metastatic relapse or metastatic progression and for real-time monitoring of cancer therapies. A number of methods for isolating CTCs have been reported, including fluorescence-activated cell sorting and magnetic-activated cell sorting. Reports of microfluidic chips have increased recently because hydrodynamic methods based on inertial microfluidics are widely applicable and thus enable continuous high-throughput separation of a variety of particles [27–29].

On the other hand, intermittent shedding of CTCs into the bloodstream and the genomic instability of malignant cells can lead to false-negative results when using prognostic strategies based simply on the presence or absence of CTCs [30–33]. Moreover, the metastatic potential of CTCs may vary [34]. Increased understanding of the high prognostic value of being able to identify CTC subsets capable of generating a metastatic deposit has led to increased interest in development of methods that will enable genomic characterization of individual CTCs and thus facilitate the rational design of targeted anticancer therapies [35, 36]. However most microfluidic chips for isolating CTCs are closed systems, so trapped CTCs from samples are more likely to be lost and recovery of isolated single CTCs is poor.

Therefore, we developed a ‘Rare cell sorter’ that utilizes a new open-channel microfluidic chip. For single-cell analysis, the open-channel microfluidic chip is designed to allow users to isolate rare cells from low-pretreated whole blood and recover the isolated cells directly. The optical system and motorized stages are packaged in the ‘Rare cell sorter’ for isolation, detection, and recovery of cells in a single unit.

In the present study, we focused on CTCs and evaluated the usefulness of the open-channel microfluidic chip with blood samples from patients with metastatic breast cancer. After recovery of isolated CTCs from the chip, we utilized a Bioanalyzer and confirmed that isolation and recovery using the ‘Rare cell sorter’ does not damage the cells. It is thought to be not only from our method of CTC isolation and recovery but also from non-pretreatment of blood sample (only dilution).

From the isolation experiments using a mouse CTC model, we confirmed collection rates of 90.6% for our open-channel microfluidic chip. Moreover, from clinical experiments using blood samples from patients with metastatic breast cancer and health volunteers, we confirmed a numerical difference in CTCs between the two groups. Furthermore, by comparing the serum CA15-3 level and number of CTCs, the usefulness of the chip was verified. The most important advantage derived from the open-channel is the ability to recover isolated CTCs directly and easily for single-cell analysis.

Until now, isolation and counting of cells were the primary objectives of CTC research. However, evidence now indicates there are differences between individual CTCs. Although molecular analysis of individual, isolated CTCs is necessary to generate tumor-specific information, this area has not been thoroughly reviewed to date, prompting increased emphasis on the development of methods for the isolation and analysis of CTCs at the single-cell level. Thus, single-cell analysis has been increasingly recognized as an important key technology. A number of sophisticated microfluidic devices have been designed to achieve single-cell analysis, including those for cell isolation, detection, and sorting (or recovery) [37]. For this study, we attempted to mainly solve the difficulty of cell recovery. Even though our isolation method is size-based and the applicable target is limited, the new concept utilizing the combination of an open-channel microfluidic chip and micropipette manipulation to achieve cell recovery is meaningful.

## Conclusions

The present paper discussed the development of a prototype device, the ‘Rare cell sorter’, for isolating and recovering single rare cells from the whole blood samples. This device had an open-channel microfluidic chip for rare cell isolation and a micropipette manipulation for single cell recovery. Even though our isolation method is size-based and the applicable target is limited, the new concept utilizing the combination of an open-channel microfluidic chip and micropipette manipulation to achieve cell recovery is meaningful. Therefore, making hybrid method for cell isolation, we hope that open-channel microfluidic chip expand the application area, such as pre-treatment for single cell analysis.

## Supporting information

S1 Fig Design of the open-channel microfluidic chip. We designed open-channel microfluidic chips with three paired values of W_P_ (distance between two micropillars) and ϕ_P_ (diameter of micropillars): (6 μm, 20 μm), (7 μm, 18 μm), and (8 μm, 16 μm). H_P_ (height of micropillars) is 30 μm, and ϕ_Pocket_ (diameter of pocket) is approximately 32 μm. The fabrication process is illustrated in S2 Fig, and results of the evaluation of each pair of W_P_ and ϕ_P_ are shown in S3 Fig.
(TIF)

S2 Fig Fabrication processes of the open-channel microfluidic chip. A mold for the poly-dimethylsiloxane (PDMS) pattern was constructed using photolithography and the mold of it was fabricated by dry etching of a silicon (Si) wafer. For the first fabrication process, a photomask was made using a laser lithographic pattern generator (DWL66FS, Heidelberg Instruments Mikrotechnik GmbH, Heidelberg, Germany). Next, epoxy-based photoresist (SU-8 3005, Microchem, Newton, MA, USA) was spin-coated to a thickness of 5 μm on a Si wafer (thickness = 525 μm) and baked before exposure. After baking, the SU-8 was exposed with the photomask using a Suss MA6 photolithography system (SUSS MicroTec AG, Garching, Germany). Following SU-8 exposure, the Si wafer was dry etched using RIE-800 (Samco, Kyoto, Japan). After the dry etching, the Si wafer was treated using C4F8 plasma for easy demolding of the PDMS. After PDMS molding, the patterned PDMS was bonded to the aspiration component. Before experimental use, the surface of the microfluidic chip was rendered hydrophilic by treatment with O_2_ plasma. To prevent interference between the microscope’s lens and the micropipette, we used an inverted microscope. Therefore, considering the autofluorescence of PDMS, a thin microfluidic chip is required, and we confirmed that the thickness of the PDMS used (10 mm) was not problematic.
(TIF)

S3 Fig Collection rates and purities of cancer cells. A preliminary experiment was carried out to determine the optimal W_P_ (distance between two micropillars) value. For the preliminary experiment, we spiked pre-counted 20±10 cells/sample of green fluorescent protein (GFP)-expressing human gastric cancer cells (GCIY-EGFPs) into human blood as CTC surrogates. The size of GCIY-EGFPs was measured using a cell counter (Luna, Logos Biosystems Inc., Korea). According to the manufacturer’s instructions, GCIY-EGFPs were stained with 0.04% trypan blue before being measured. The GCIY-EGFPs were found to be 15±16 μm in diameter and were thus suitable surrogates for CTCs, which are generally 15±10 μm in diameter. The results of the experiment are shown in S3 Fig 3. Nearly 90% of the GCIY-EGFPs were trapped on microfluidic chips with a W_P_ of 6 or 7 μm. However, a W_P_ of 8 μm seemed too wide for trapping GCIY-EGFPs. Also, 99.75% WBCs were removed from blood and high capture purity (~60%) was achieved in their microfluidic chip. Here, purity is the fraction of target cells relative to the total captured cells on the open-channel microfluidic chip. With a W_P_ of 7 μm we could trap many GCIY-EGFPs and remove many WBCs; thus, 7 μm was found to be the most efficient W_P_, and this distance was used for the main experiment.
(TIF)

S1 Movie Movie of CTCs isolation on the open-channel microfluidic chip. This movie shows the CTCs (green) isolation process at the meniscus of the air–liquid interface (dotted line). There is a liquid layer of blood above the dotted line and an air layer below the dotted line. On the liquid layer, the blood sample flows not only in the channel but also on the chip. On the air layer, the blood sample flows in the channel. The CTCs and blood cells are carried to the meniscus of the air–liquid interface via the drag force induced by a syringe pump and deposited into pockets of the open-channel microfluidic chip by capillary force. CTCs, which are relatively larger than other blood cells, are trapped in the pockets, and blood cells are removed as they pass thorough the gaps between the micropillars.
(MP4)

S2 Movie Movie of single CTC recovery by micropipette manipulation. This movie shows the CTC recovery (pick-up) process. Using the auto detection feature of the ‘Rare cell sorter’, CTCs are detected and some information can be obtained (e.g., circularity). Selected CTCs are recovered by a micropipette manipulation.
(MP4)

S1 Checklist NC3Rs ARRIVE guidelines checklist. (PDF)
