# Supplementary figures and images for: Rare cell isolation and recovery on open-channel microfluidic chip
Source: PLoS One. 2017 Apr 20;12(4):e0174937. doi: 10.1371/journal.pone.0174937 (PMC5398523; doi:10.1371/journal.pone.0174937)

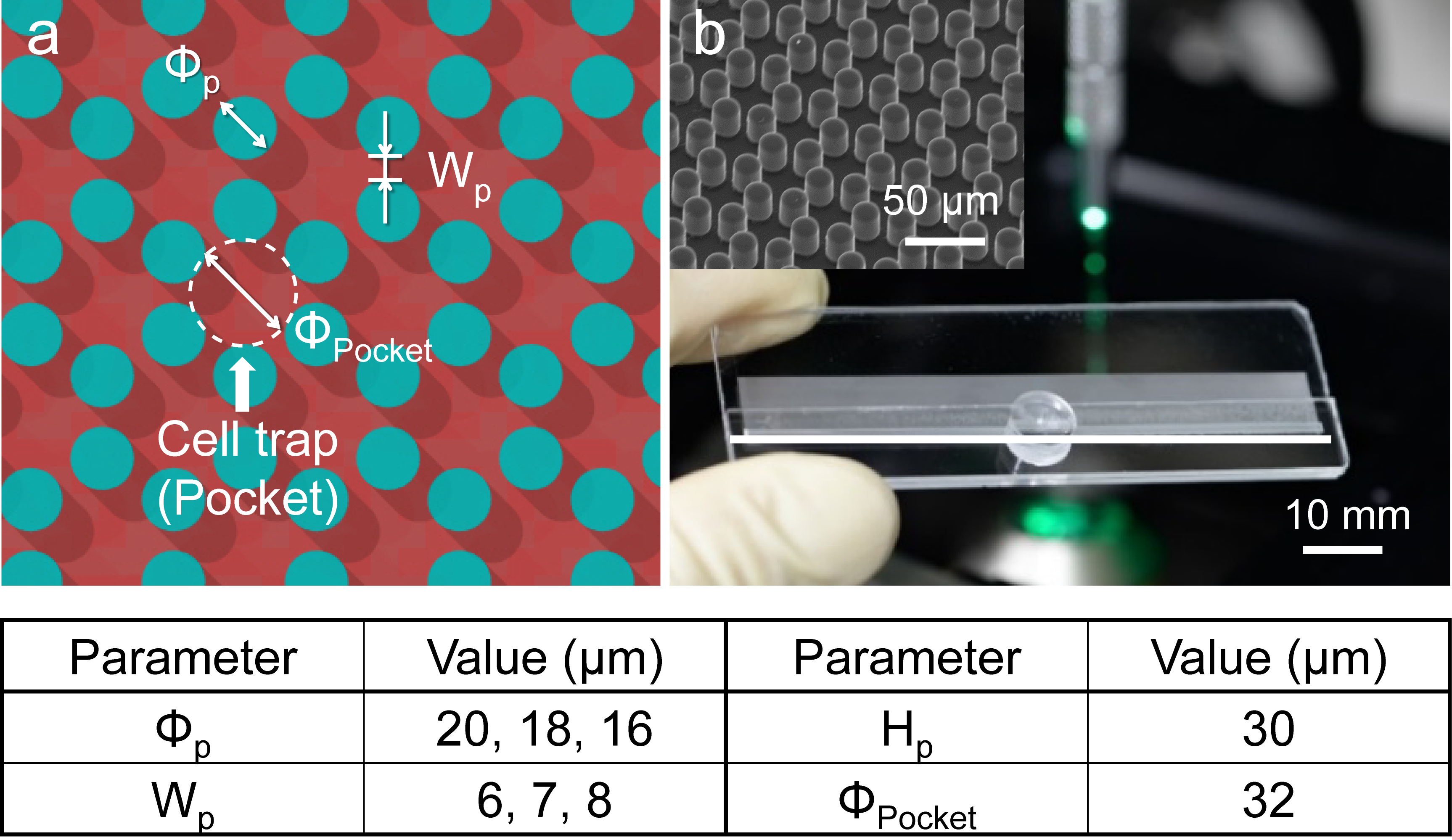

Supplement: S1 Fig — We designed open-channel microfluidic chips with three paired values of WP (distance between two micropillars) and ϕP (diameter of micropillars): (6 μm, 20 μm), (7 μm, 18 μm), and (8 μm, 16 μm). HP (height of micropillars) is 30 μm, and ϕPocket (diameter of pocket) is approximately 32 μm. The fabrication process is illustrated in S2 Fig, and results of the evaluation of each pair of WP and ϕP are shown in S3 Fig. (TIF) [file pone.0174937.s001.tif]

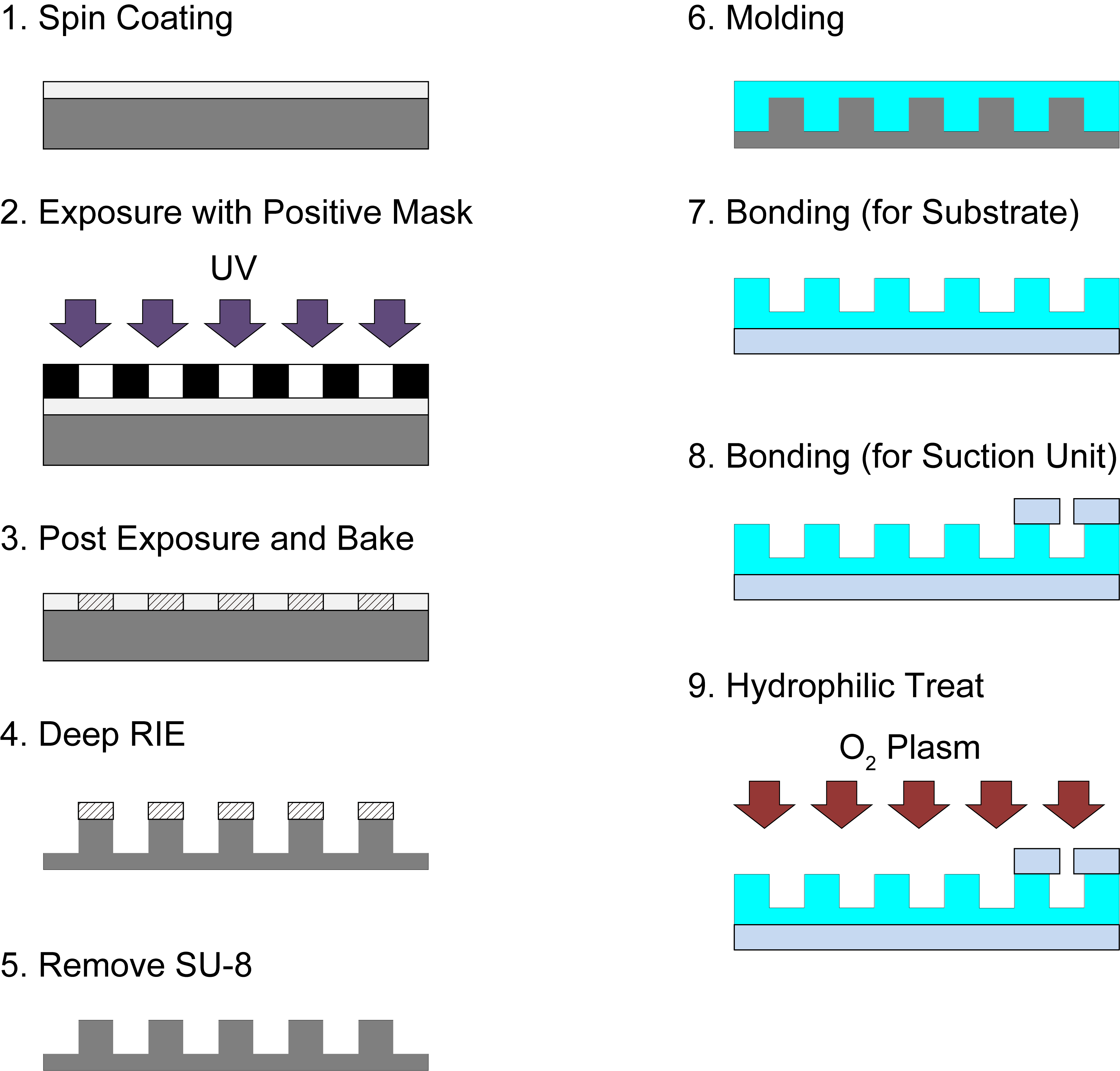

Supplement: S2 Fig — A mold for the poly-dimethylsiloxane (PDMS) pattern was constructed using photolithography and the mold of it was fabricated by dry etching of a silicon (Si) wafer. For the first fabrication process, a photomask was made using a laser lithographic pattern generator (DWL66FS, Heidelberg Instruments Mikrotechnik GmbH, Heidelberg, Germany). Next, epoxy-based photoresist (SU-8 3005, Microchem, Newton, MA, USA) was spin-coated to a thickness of 5 μm on a Si wafer (thickness = 525 μm) and baked before exposure. After baking, the SU-8 was exposed with the photomask using a Suss MA6 photolithography system (SUSS MicroTec AG, Garching, Germany). Following SU-8 exposure, the Si wafer was dry etched using RIE-800 (Samco, Kyoto, Japan). After the dry etching, the Si wafer was treated using C4F8 plasma for easy demolding of the PDMS. After PDMS molding, the patterned PDMS was bonded to the aspiration component. Before experimental use, the surface of the microfluidic chip was rendered hydrophilic by treatment with O2 plasma. To prevent interference between the microscope’s lens and the micropipette, we used an inverted microscope. Therefore, considering the autofluorescence of PDMS, a thin microfluidic chip is required, and we confirmed that the thickness of the PDMS used (10 mm) was not problematic. (TIF) [file pone.0174937.s002.tif]

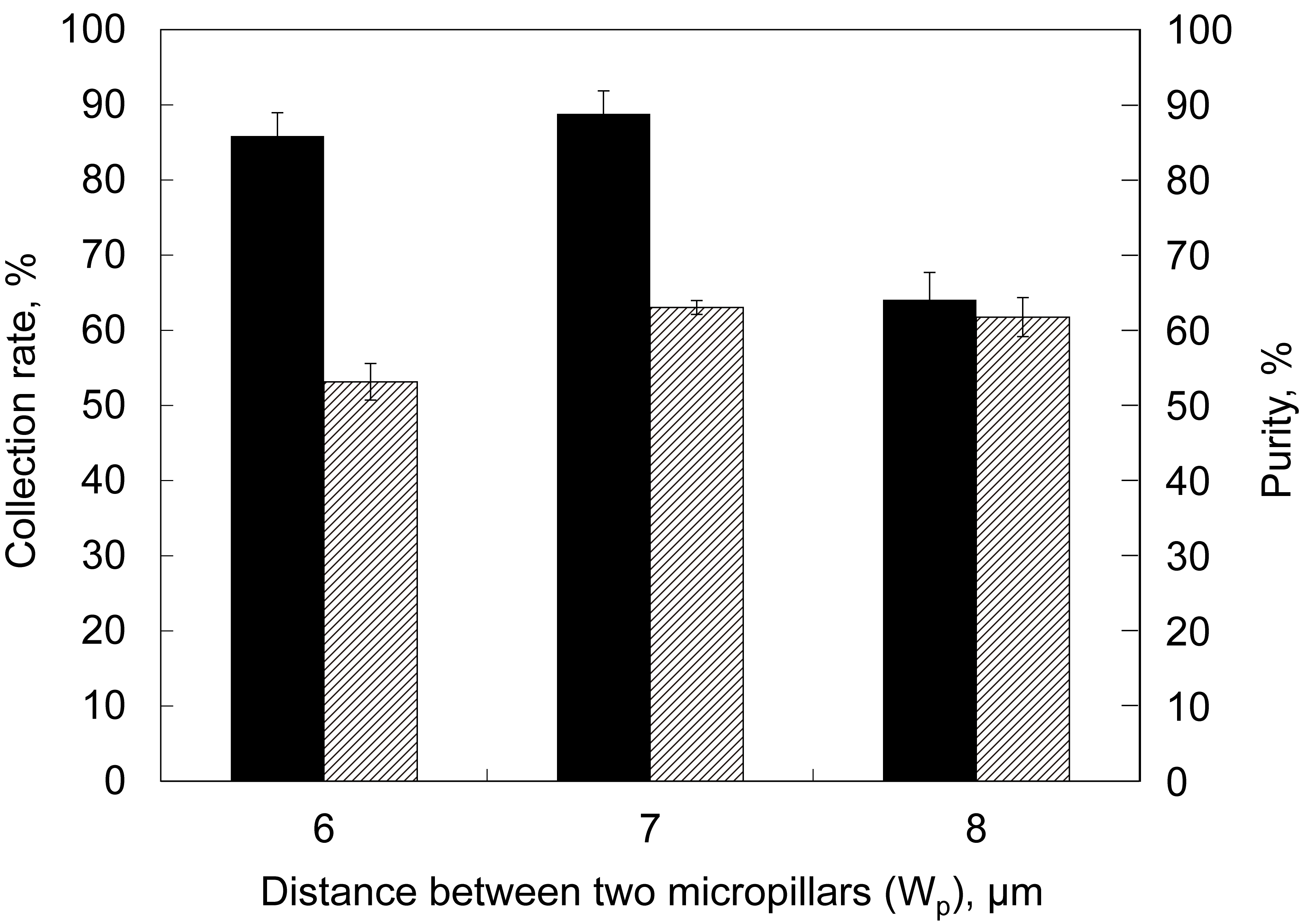

Supplement: S3 Fig — A preliminary experiment was carried out to determine the optimal WP (distance between two micropillars) value. For the preliminary experiment, we spiked pre-counted 20±10 cells/sample of green fluorescent protein (GFP)-expressing human gastric cancer cells (GCIY-EGFPs) into human blood as CTC surrogates. The size of GCIY-EGFPs was measured using a cell counter (Luna, Logos Biosystems Inc., Korea). According to the manufacturer’s instructions, GCIY-EGFPs were stained with 0.04% trypan blue before being measured. The GCIY-EGFPs were found to be 15±16 μm in diameter and were thus suitable surrogates for CTCs, which are generally 15±10 μm in diameter. The results of the experiment are shown in S3 Fig 3. Nearly 90% of the GCIY-EGFPs were trapped on microfluidic chips with a WP of 6 or 7 μm. However, a WP of 8 μm seemed too wide for trapping GCIY-EGFPs. Also, 99.75% WBCs were removed from blood and high capture purity (~60%) was achieved in their microfluidic chip. Here, purity is the fraction of target cells relative to the total captured cells on the open-channel microfluidic chip. With a WP of 7 μm we could trap many GCIY-EGFPs and remove many WBCs; thus, 7 μm was found to be the most efficient WP, and this distance was used for the main experiment. (TIF) [file pone.0174937.s003.tif]
